# Development and Validation of Adherence Score for Subcutaneous Biologic Disease-Modifying Antirheumatic Drugs

**DOI:** 10.3389/fphar.2020.572260

**Published:** 2020-11-09

**Authors:** Salmi Abdul Razak, Mohd Makmor Bakry, Mohd Shahrir Mohamed Said, Chai-Eng Tan, Adyani Md Redzuan

**Affiliations:** ^1^ Faculty of Pharmacy, Universiti Kebangsaan Malaysia, Kuala Lumpur, Malaysia; ^2^ Hospital Tuanku Ja'afar Seremban, Seremban, Malaysia; ^3^ Department of Medicine, UKM Medical Centre, Kuala Lumpur, Malaysia; ^4^ Faculty of Medicine, Universiti Kebangsaan Malaysia, Kuala Lumpur, Malaysia

**Keywords:** instrument development, content validity, questionnaire, medication adherence, biologic disease-modifying antirheumatic drugs

## Abstract

**Background**: The biologic disease-modifying antirheumatic drugs (bDMARDs) are currently incorporated as part of the pharmacotherapy management of inflammatory arthritis (IA). Adherence to bDMARDs is crucial to ensure treatment success in IA. However, most of the recent studies evaluated adherence level in patients using subcutaneous injections of bDMARDs utilized the indirect methods adapted from adherence assessment for oral medication.

**Aim**: This study aimed to develop a questionnaire to assess adherence to the self-injectable subcutaneous bDMARDs.

**Methods**: The development of the Subcutaneous bDMARDs Adherence Score (SCADS) involved evaluation of content validity. Literature reviews provide the basis for domain identification and item formation. Four experts evaluated the instrument by using a four-point ordinal scale with a rubric scoring on relevance, importance, and clarity of each item in measuring the overarching construct. The item-level content validity index (I-CVI) and the scale-level content validity index (S-CVI) were calculated. The factor structure and internal consistency reliability of SCADS were estimated using principal component analysis (PCA) and Cronbach’s alpha, respectively.

**Results**: Both S-CVI/UA (universal agreement) and the average item-level content validity index (S-CVI/Ave) (average) for the entire instrument showed excellent criteria with a value of >0.90. Cronbach’s alpha coefficient value for SCADS was 0.707 indicating good internal consistency. All items showed corrected item-total correlation coefficients above 0.244. Questionnaire items with a factor loading of 0.30 or above were considered in the final factor solution. The factor analysis resulted in 3-factor solutions, which corresponded to 66.62% of the total variance.

**Conclusion**: The SCADS is a consistent and reliable instrument for evaluating adherence among IA patients using the subcutaneous bDMARDs. It is simple to use, yet comprehensive but still requiring further clinical and international validation.

## Introduction

Inflammatory arthritis (IA) is a chronic autoimmune disease characterized by inflammation of the joints and other tissues. Common types of IA include rheumatoid arthritis (RA), spondyloarthropathies such as ankylosing spondylitis (AS), axial spondyloarthritis (AxSpA), and peripheral spondyloarthritis (pSpA), psoriatic arthritis (PsA), juvenile idiopathic arthritis (JIA), and systemic lupus erythematosus (SLE) (Sandler and Dunkley, 2018). Amongst all, RA is the most common chronic systemic autoimmune disease with an estimated prevalence of 1% of the world population (Shahrir et al., 2008; Rajalingham, 2018). These IA are chronic diseases that involve long-term pharmacotherapy management. The therapeutic goals of IA are to achieve good control of the disease activity, to reduce the progression of permanent joint damage, and to improve patients’ quality of life. Pharmacotherapy modalities in the management of IA include the use of the conventional synthetic disease-modifying antirheumatic drugs (csDMARDs) followed by the targeted biologic disease-modifying antirheumatic drugs (bDMARDs) (Smolen et al., 2014; Razak et al., 2018). The DMARDs are the key agents recommended in the management for IA disease control, damage prevention, persevering affected joint function, increase in patients’ quality of life, and increase in the possibilities to achieve complete remission of the disease (Wee et al., 2016). Nonsteroidal anti-inflammatory drugs (NSAIDs) and low-dose systemic corticosteroids are usually prescribed. The NSAIDs may help to reduce pain and inflammation presented as swelling, whereas the short-term use of low-dose systemic corticosteroid is often viewed as bridging therapy in managing flares and as part of the initial treatment strategy in the management of early rheumatoid arthritis (Smolen et al., 2014; Kavanaugh and Wells, 2014).

However, the benefits of the pharmacotherapy management and treatment goals of IA will not be optimized if patients do not adhere to the medication prescribed. The impact of therapeutic nonadherence among the IA patients includes premature discontinuation of effective therapy, increased immunogenicity of bDMARDs, poor disease outcome, reduction of quality of life, and increase in health utilization cost. Invasive nonpharmacological treatment modalities such as surgery are required to manage such damage involving structural and radiological progression (Marengo and Suarez-Almazor, 2015).

The European League Against Rheumatism (EULAR) recommendation in the pharmacotherapy management of RA follows the treat-to-target strategies. These strategies include either to increase the drug dosages, to add or to switch therapeutic agents with an aim to lower the disease activity, leading to disease remission (Smolen et al., 2016). To ensure the strategies are successful, monitoring of patients’ adherence to medication is crucial. Implementing treat-to-target strategies without considering patient’s adherence level toward bDMARDs prescribed may result in irrational prescribing, increases the costs in drug utilization, and reduces the efficacy of the medication leading to increased disability and disease progression.

Assessment of medication adherence can be done by using either direct or indirect methods. The direct methods include conducting biologic assays on measuring the drug concentration levels, or direct observation upon ingestion of medications. The indirect methods for medication adherence assessments include pharmacy prescription refill rates, patient interviews, pill counts, patients diaries, medication event monitoring system (MEMS), and self-reported questionnaires (Marengo and Suarez-Almazor, 2015; Curtis et al., 2016). Indirect methods are preferred compared to direct methods as these methods are noninvasive, readily available, less costly, and practical.

Indirect methods of adherence assessment on oral csDMARDs have been extensively discussed, but few were conducted on the subcutaneous bDMARDs. Most of the studies predicting nonadherence to subcutaneous bDMARDs therapy were done by assessing the persistence rates of bDMARDs or the rate of discontinuation of bDMARDs (Blum et al., 2011; Neubauer et al., 2014; Lyu et al., 2016; Mahlich and Sruamsiri, 2016; Calip et al., 2017). The Morisky Medication Adherence Scale (MMAS) and the Compliance Questionnaire on Rheumatology (CQR) are the currently available validated questionnaires. However, both of the questionnaires measure adherence related to oral medications, but not specifically to self-injectable subcutaneous bDMARDs (Hughes et al., 2013; Cheong et al., 2015). The current EULAR recommendations in the management of RA with csDMARDs and bDMARDs did emphasize that aspirational in nature was considered in constructing the guideline recommendations despite evidence-driven point of view. One of the aspirational recommendations indicated that patients were assumed to adhere to their medication. However, no exact method was provided or suggested to measure patients’ adherence to the bDMARDs (Smolen et al., 2017; Smolen et al., 2020). A simple tool to assess the adherence status of patients on subcutaneous bDMARDs is therefore invaluable for both clinical and research use. This study reports the development of a new brief medication adherence tool, specifically for the self-injectable subcutaneous bDMARDs prescribed among IA patients.

## Materials and Methods

This study involved two major stages of questionnaire development namely the development stage and the judgement-quantification stage (Lynn, 1986). The development stage involved domain identification and item generation of the questionnaire to evaluate patient’s adherence to the subcutaneous bDMARDs. This is followed by the second stage (judgement-quantification) to evaluate the content validity of the developed questionnaire (Boateng et al., 2018). Evaluation of content validity was done thoroughly to investigate whether the items in the research instrument or tool represent the measured outcome. Ethical approval for this research has been granted from the Research Ethics Committee, Universiti Kebangsaan Malaysia (ETHICS COMMITTEE/IRB REF NO: UKM PPI/111/8/JEP-2017-059).

### Stage 1: Development

The development stage includes domain identification and item generation. Domain identification for this study was carried out based on the literature reviews, which focused on medication adherence measurements (Lynn, 1986; Aravamudhan and Krishnaveni, 2015; Zamanzadeh et al., 2015). The developed questionnaire consists of one main domain, which was adherence. The items of the questionnaire were developed based on the literature review, which focused on barriers and facilitators to medication adherence in chronic diseases, oral csDMARDs, and bDMARDs (Borah et al., 2009; Li et al., 2010; Bluett et al., 2015; Salaffi et al., 2015; Voshaar et al., 2016; Bhoi et al., 2017; Smolen et al., 2019). The questionnaire focused on 1) bDMARDs medication-taking behavior, which was defined as the way that the patient behaves or acts or conducts oneself in a particular situation, especially toward medication-taking of the bDMARDs administration, and 2) patient’s knowledge and clarity of information given by the patient about the current dosage of the subcutaneous bDMARDs. The items for the Subcutaneous biologic disease-modifying antirheumatic drugs (bDMARDs) Adherence Score (SCADS) questionnaire require patients to answer each item by answering in either a “yes/no” or a “right/wrong” format. This dichotomous binary response format is preferred to confirm that there is no scope of middle perspective for each question answered, to promote a definite interpretation and easier application of the instrument. Sentence structures of these items were refined to clearly design interrogative sentence, avoiding patients answering questions in the affirmative. A Malay language version was developed in view of respondents’ native language that will increase respondents’ comprehension of each item developed in the SCADS instrument. Six versions of the SCADS instrument were subjected to face validity, to assess whether the items designed in the questionnaire appear validly measuring the variable or the construct, as intended. A summary of content development for all six versions of the questionnaire is presented in Table 1. For the purpose of publication, all items in the questionnaire were translated into English by a bilingual language expert.

**TABLE 1:** Description of items included in the development of SCADS (final version 1.6).

Construct	Item no.	Description of item	Vers 1.6	Vers 1.5	Vers 1.4	Vers 1.3	Vers 1.2	Vers 1.1
1. Knowledge and clarity of information on current dosage of bDMARDs	Q1	Admitting injecting bDMARDs following the prescribed dose	/	/	/	/	/	/
	Q2	Verifying strength of bDMARDs taken	/	/	/	/	/	x
	Q3	Claiming to inject bDMARDs according to prescribed frequency	/	/	/	x	x	x
	Q4	Verifying frequency of bDMARDs injection taken	/	x	x	x	x	x
	Q5	Claiming to inject bDMARDs according to specific day or date	/	/	/	x	x	x
	Q6	Verifying the specific day or date of bDMARDs injection taken	/	/	x	x	/	/
2. Medication-taking behavior on bDMARDs	Q7	Forgetfulness in injecting bDMARDs	/	/	/	/	/	/
	Q8	Continue injecting bDMARDs even though feeling better with no joint pain	/	/	/	/	/	/
	Q9	The commitment of having bDMARDs injection when traveling abroad	/	/	/	/	/	/
	Q10	Temporary discontinuation of bDMARDs when having an infection	/	/	/	/	/	/

### Stage 2: Judgement-Quantification

Content validity is defined as the extent to which the elements or items within a measurement procedure are relevant and representative of the constructs intended to be measured (Lynn, 1986; Haynes et al., 1995). An expert panel was formed to assess the content validity of the new tool. The panel comprised of two academicians from the field of pharmaceutical and clinical pharmacy, a consultant rheumatologist, and a practicing pharmacist who was credentialed and appointed as preceptor of the Rheumatology Medication Therapy Adherence Clinic (R-MTAC). In the Malaysian setting, the R-MTAC is a pharmacist-led medication therapy management in rheumatology clinic, which provides education on IA diseases and skills on self-injecting bDMARDs, as well as empowering medication adherence related to DMARDs therapy among IA patients. Pharmacists involved in R-MTAC would also assist the prescribers on drug information, pharmacotherapy consults, and availability of DMARDs in daily clinical practices.

All appointed experts in this study had knowledge and experience relevant to the aim of the questionnaires. The expert panel thoroughly evaluated all items generated. They were assessed in terms of relevance, importance, and clarity in measuring the overarching construct based on the 4-point scale. Item which was not relevant or not necessary to measure the construct or not clear was graded as one point, whereas item which was clear, relevant, or essential to measure the construct was graded as four points. The expert panel also provided comments and suggestions for item improvement.

### Evaluation of Content Validity and Reliability

The content validity index of individual items (I-CVI) was defined as the proportion of content experts giving the item a relevance rating of three or four based on the 4-point rating scale (Waltz and Bausell, 1981; Lynn, 1986). For a new instrument, an item was considered appropriate, with an I-CVI value of more than 0.80 (Davis, 1992). Items with I-CVI value higher than 0.79 were considered appropriate; items with a range of I-CVI values between 0.70 and 0.79 were further revised or rephrased for a meaningful cohesion; and if the I-CVI value was less than 0.70, the particular item was suggested to be eliminated. Based on the number of expert judges involved in the quantification of content validity, the I-CVI value of 1.00 was needed for expert judges of a minimum of three to five raters, and for the number of expert judges of six or more raters, an I-CVI value of more than 0.78 was considered acceptable (Lynn, 1986).

The next step involved the evaluation of the average content validity index for scale (S-CVI/Ave) and universal agreement content validity index for scale (S-CVI/UA). The recommended acceptable value for S-CVI/UA was at least 0.80, while for the S-CVI/Ave, the standard for the index of average congruity of at least 0.90 was required (Davis, 1992; Waltz et al., 2017). For face validation, the preliminary questionnaire underwent five review sessions with the expert panel to assess for comprehensibility and its acceptability. Double-barrel questions were removed and rephrased. Feedbacks from the respondents were used to amend the items to obtain the most accurate wording for the questionnaire.

After face validation, the final version of the SCADS questionnaire was then administered to 50 patients for feasibility testing (Table 6). The internal consistency reliability of the questionnaire was estimated by Cronbach’s alpha coefficient and the corrected item-total correlations. A Cronbach’s alpha coefficient value greater than or equal to 0.70 was considered acceptable (Nunnally and Bernstein, 1994). The corrected item-total correlations estimate how well the item scores were correlated to the total score of the questionnaire or domain (Priest et al., 1995). Items with a corrected item-total correlation of more than 0.2 were considered to be acceptable, indicating that the items are measuring the same underlying concept (Mikhael et al., 2019).

Exploratory factor analysis (EFA) was conducted to explore the possible underlying factor structure or dimensions among variables in SCADS. The number of factors extracted was determined by Kaiser’s eigenvalue equal to or greater than 1.0. Average communalities of more than 0.6 indicate a good correlation with all items. Factor loadings of ≥0.30 on each item were considered belonging to the corresponding factors and confirm the construct validity of the questionnaire (Field, 2013). The number of components to be retained was assessed based on a scree plot. Components above the elbow shape of the plot were retained (Cattell, 1966). Statistical analysis was performed using the IBM SPSS software version 25.0.

## Results

A total of 50 IA patients were included for feasibility testing of the SCADS tool. There were 42 (84%) female patients with a mean age of 45.5 ± 11.7 years. Majority of the patients were diagnosed with rheumatoid arthritis (RA) (n = 29, 58%). Patients’ characteristics are described in Table 2.

**TABLE 2:** Demographic profile of sample population for SCADS.

	Total n = 50 (100%)
Age (years) (mean SD)	45.5 ± 11.7
Gender	
Male	8 (16%)
Female	42 (84%)
Ethnicity	
Malay	32 (64%)
Chinese	4 (8%)
Indian	14 (28%)
Type of inflammatory arthritis (IA)	
Ankylosing spondylitis	8 (16%)
Psoriatic arthritis	13 (26%)
Rheumatoid arthritis	29 (58%)
Subcutaneous bDMARDs currently used	
Adalimumab	10 (20%)
Etanercept	14 (28%)
Golimumab	10 (20%)
Tocilizumab	14 (28%)
Secukinumab	2 (4%)

Based on the expert panel evaluation, the S-CVI/UA and S-CVI/Ave values were >0.90, indicating excellent agreement on relevance of the items by all raters. The overall internal consistency reliability for the SCADS questionnaire with ten items was 0.707. Corrected item-total correlation values ranged between 0.244 and 0.590. Table 3 displays Cronbach’s alpha coefficient values for the overall SCADS questionnaire and the corrected item-total correlation for each item.

**TABLE 3:** Corrected item-total correlation for items in SCADS.

Item No	Description of item	SCADS with ten (10) items	
Overall Cronbach’s alpha (α) = 0.707		Corrected item-total correlation	Cronbach’s alpha (α) if item deleted
Q1	Admitting injecting bDMARDs following the prescribed dose	0.281	0.699
Q2	Verifying strength of bDMARDs taken	0.306	0.698
Q3	Claiming to inject bDMARDs according to prescribed frequency	0.590	0.650
Q4	Verifying frequency of bDMARDs injection taken	0.590	0.650
Q5	Claiming to inject bDMARDs according to specific day or date	0.500	0.665
Q6	Verifying the specific day or date of bDMARDs injection taken	0.500	0.665
Q7	Forgetfulness in injecting bDMARDs	0.300	0.696
Q8	Continue injecting bDMARDs even though feeling better with no joint pain	0.286	0.696
Q9	The commitment of having bDMARDs injection when traveling abroad	0.244	0.707
Q10	Temporary discontinuation of bDMARDs when having an infection	0.283	0.713

**FIGURE 1:**
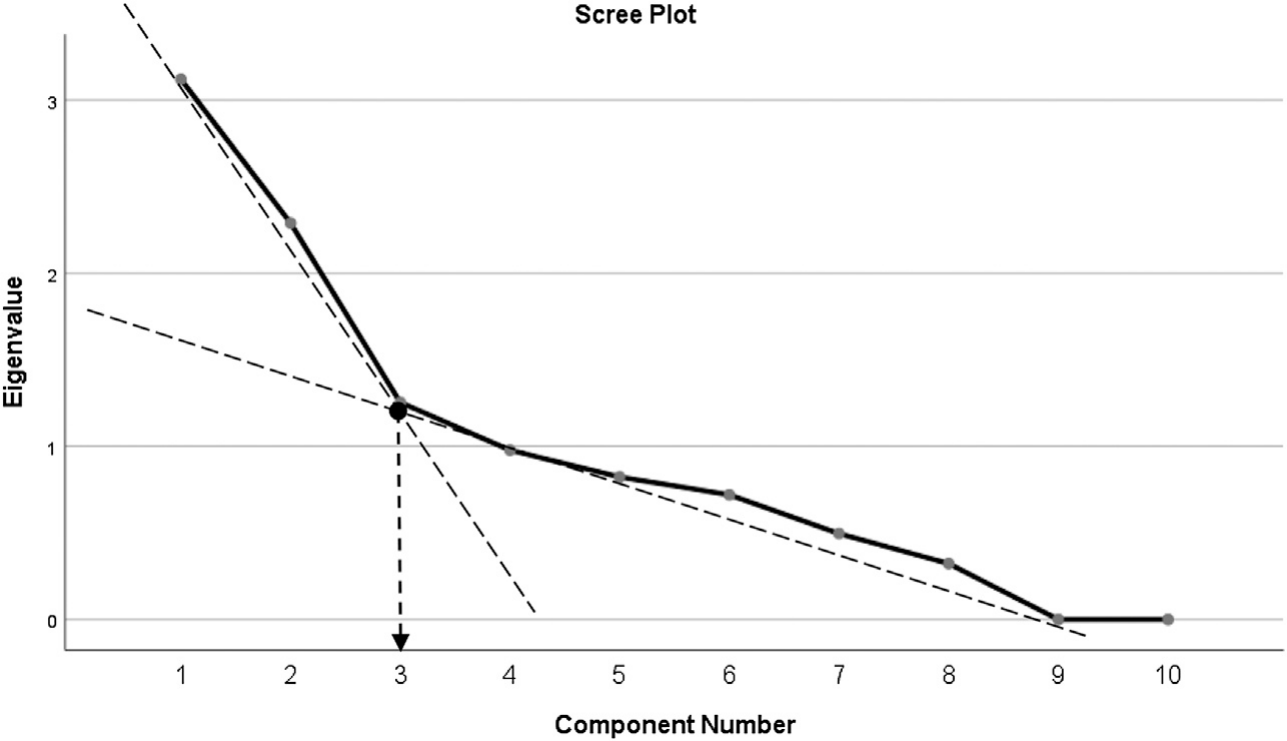
Scree plot of factor analysis of SCADS.

Principal component analysis (PCA) revealed communalities ranging from 0.316 to 0.924, with an average communality of 0.666. A total of three components with eigenvalues exceeding one (1) were extracted, with variances explained of 31.12% (for the first component), 22.89% (second component), and 12.53% (third component), respectively. The three-component or factor solution explained a total of 66.62% of the variance. Upon visual inspection of the scree plots, three factors or components were retained (Figure 1). A simple structure was obtained by Oblimin rotation. All items show loadings of more than 0.30 (Table 4).

**TABLE 4:** The pattern and structure matrix for PCA of the three-component solution of SCADS items.

Item no.	Pattern matrix^a^			Structure matrix			Communalities
	Component			Component			
	1	2	3	1	2	3	
Q4	**0.956**			**0.954**			0.924
Q3	**0.956**			**0.954**			0.924
Q2	**0.827**			**0.798**			0.698
Q6		**0.969**			**0.944**		0.904
Q5		**0.969**			**0.944**		0.904
Q7		**0.622**			**0.626**		0.412
Q8		**0.395**			**0.461**		0.321
Q1			**-0.707**			**0.687**	0.741
Q9			**0.653**			**-0.622**	0.519
Q10			**0.404**			**0.438**	0.316
Eigenvalue	3.120	2.289	1.253	3.120	2.289	1.253	
% of variance	31.196	22.889	12.532	31.196	22.889	12.532	

Extraction method: principal component analysis (PCA).

Rotation Method: Oblimin with Kaiser normalization.

a Rotation converged in nine iterations.

The internal reliability coefficient for SCADS with ten questions was 0.707. Component 1 and component 2 were found to have a good internal reliability coefficient value of 0.875 and 0.753, respectively. However, Cronbach’s alpha for component 3 revealed a low alpha value (α = 0.091) (Table 5). Removing items Q1, Q9, and Q10 from the SCADS scale would generate a Cronbach’s alpha value of 0.704 for the overall questionnaire, which is less than the internal reliability coefficient for SCADS with ten questions (α = 0.707). Since deletion of any of the three items in component 3 would not improve the internal reliability coefficient value, the total questions to be included in the final questionnaire were retained as ten questions. Version 1.6 represented the final version of the questionnaire for the SCADS tool (Table 6).

**TABLE 5:** Reliability test and corrected item-total correlation of SCADS questionnaire based on the three-component solution obtained.

Component	Item		Corrected item-total correlation	Cronbach’s alpha value (α)
Alpha value of overall questionnaire = 0.707				
Component 1	Q4	Verifying frequency of bDMARDs injection taken	0.867	0.875
	Q3	Claiming to inject bDMARDs according to prescribed frequency	0.867	
	Q2	Verifying strength of bDMARDs taken	0.628	
Component 2	Q6	Verifying the specific day or date of bDMARDs injection taken	0.765	0.753
	Q5	Claiming to inject bDMARDs according to specific day or date	0.765	
	Q7	Forgetfulness in injecting bDMARDs	0.405	
	Q8	Continue injecting bDMARDs even though feeling better with no joint pain	0.342	
Component 3	Q1	Admitting injecting bDMARDs following the prescribed dose	-0.068	0.091
	Q9	The commitment of having bDMARDs injection when traveling away from home	0.076	
	Q10	Temporary discontinuation of bDMARDs when having an infection	0.103	

**TABLE 6:** Subcutaneous biologic disease-modifying antirheumatic drugs (bDMARDs) Adherence Score (SCADS**)** version 1.6.

Item number	Item
Q1	Do you administer (inject) your biologic injection as the prescribed dose for each biologic injection session?
Q2	Write the strength of your biologic injection dose; for example, the strength in “milligram (mg)” or write the number of biologic injections you are taking; for example, 1 @ 2 @ 3 injections taken at each biologic injection session
Q3	Do you administer (inject) your biologic injection according to the prescribed frequency within a period of time?
Q4	Write how many times the injection must be taken within a period of time; for example, once a month, twice a month, or every 4 weeks
Q5	Do you administer (inject) your biologic injection on a specific day or date that you must take (inject) your biologic injection?
Q6	Write the specific day or write the specific date of the month that you must take (inject) your biologic injection
Q7	Did you ever forget to do your biologic injection within the past 1 (one) month?
Q8	Do you continue taking (injecting) your biologic injection even though you feel better and have no joint pain?
Q9	Do you bring along your biologic injection when you travel away from home? For example, traveling for long holidays, attending social events such as wedding reception, feast, and party.
Q10	Do you stop temporarily from taking (injecting) your biologic injection dose when you feel unwell? For example, having a fever, or coughing with phlegm, or taking antibiotics

## Discussion

This study demonstrates the accomplishment of developing a new tool to measure the adherence status among IA patients taking the subcutaneous bDMARDs. Compared to other retrospective indirect methods to assess adherence such as the Medication Possession Ratio (MPR) or to predict drug survival and persistence rates through databases, SCADS offers for a prospective assessment of drug adherence measurement in daily routine clinical practice. Generating the items in the newly developed questionnaire was done based on literature reviews and expert opinions. Items included in SCADS were based on the underlying theory of failure to adhere to a medication regimen or omitting medication doses, in which both occurred due to forgetfulness or discontinuing medication when feeling better (Morisky et al., 1986). The underlying theory used in generating items in SCADS was similar to the Morisky Medication Adherence Score (MMAS) for self-reporting adherence measure in hypertensive patients (Morisky et al., 1986; Morisky et al., 2008). Compared with MMAS, the SCADS questionnaire was tailored for IA patients using self-injecting bDMARDs.

Prior to this questionnaire development, self-reporting adherence measures that were widely used for rheumatic diseases include the 19-item version of the Compliance Questionnaire for Rheumatology (CQR19) and the 5-item version of the Compliance Questionnaire for Rheumatology CQR5 (Klerk et al., 1999; Hughes et al., 2013). The four-point Likert scale was used for both questionnaires with answering scale scored one (1) for “Definitely don’t agree,” to score four (4) for “Definitely agree.” The CQR19 was firstly developed for rheumatoid arthritis (RA), polymyalgia rheumatica (PMR), and gout patients taking oral antirheumatic medication. The lengthiness of the CQR19 was then reduced by using only five (5) questions from CQR19, which then named the CQR5. Both had an internal reliability of 0.71 and 0.85, respectively (Klerk et al., 1999; Klerk et al., 2003; Hughes et al., 2013). However, the items in CQR were developed as statements to detect possible reasons on barriers to oral antirheumatic medication compliance, rather than an assessment of medication-taking behavior toward adherence to the subcutaneous bDMARDs as developed in SCADS.

The items included in SCADS focused on the assessment of medication-taking behavior, together with knowledge of the current dose, strength, and frequency of the bDMARDs. Patients answering “yes” to Q3 and Q5 regarding the prescribed dose, strength, and frequency or timing of the bDMARDs were subjected to verification by the attending pharmacist. This process involved the pharmacist assessing through the patient’s latest medical record and endorses the accuracy of the information provided by the respondent. This technique provides a confirmation for each item assessed and yet offers simplicity for target responders.

All the items in the SCADS questionnaire were evaluated for relevance, importance, and clarity by the expert judges following the method suggested by Lynn (1986). This method is widely applied in health care researches as well as in social sciences. It is considered as a requirement needed in the first stage of questionnaire development in conducting the validation study (Lynn, 1986; Haynes et al., 1995; Wynd et al., 2003; Polit and Beck, 2006). The I-CVI, S-CVI/UA, and S-CVI/Ave for each item and the overall questionnaire of the SCADS instrument were interpreted as having excellent content validity with the value of >0.90. Apart from this process, all written and verbal comments from the expert judges were taken into consideration while making the decisions of either to remove, to add, or to rephrase the items. All the items were judged as relevant and representative of the constructs studied.

The feasibility study of the SCADS was done among 50 patients who were currently using their subcutaneous bDMARDs for the treatment of IA. The internal consistency reliability of the SCADS questionnaire was evaluated. Based on the psychometric principles, if the reliability of a scale or measure is achieved, it can be considered as valid (Abu Samra et al., 2016). The SCADS questionnaire with ten (10) items has good internal consistency reliability with a Cronbach’s alpha coefficient value of 0.707. Compared to the past studies using binary responses in measuring medication-taking behavior for adherence in medicine, the MMAS-4 and MMAS-8 had Cronbach’s alpha coefficient values of 0.61 and 0.64, respectively. The corrected item-total correlation for each item in SCADS was more than 0.2, indicating that all ten (10) items are well correlated with the total score of the questionnaire set.

Factor analysis by PCA revealed three components which encompassed 66.62% of the total data variance. The first component of the factor analysis includes items Q2, Q3, and Q4, which describes more on awareness and knowledge of the current dose of the self-injecting bDMARDs. The items involve claiming and verifying the current frequency and strength of the bDMARDs taken by the patient. The second component of the factor analysis describes more on memorization capabilities in medication-taking behavior. This component includes 1) either the patient remembers or is able to specify the day or date to self-inject the bDMARDs, 2) if the patient had forgotten to self-inject bDMARDs within the past one month, and 3) either the patient continues or stops taking bDMARDs when the patient feels better with no joint pain. The later item (item Q8) is included in the SCADS questionnaire based on the understanding that feeling better and disappearance of unwanted symptoms are factors associated with medication nonadherence (Kardas et al., 2013).

The third component revealed the commitment of taking the self-injecting bDMARDs. Item Q1 assessed the overall commitment of knowing the dose that should be taken each time the patient injects or administers the subcutaneous bDMARDs. Further assessment was done in the following items: Q2, Q3, Q4, Q5, and Q6. Item Q9 questioned on their medication-taking behavior on the commitment to bring along their subcutaneous bDMARDs during a long vacation or traveling abroad. Upon face validity among respondents on answering the SCADS questionnaire, two respondents did subjectively comment on item Q9. They mentioned that they avoid bringing along their subcutaneous bDMARDs during a long vacation, and especially when traveling abroad. Avoidance was due to their own experience of having problems with the authorities while traveling across international borders. Rather than bringing along their subcutaneous bDMARDs, arrangements were made by the patients after acknowledging their attending rheumatologist. Such arrangements involved rescheduling their injection or avoiding traveling on the scheduled day or date that they must administer their subcutaneous bDMARDs.

In terms of the dosing interval of the subcutaneous bDMARDs, due to their longer half-life properties, the administration of the subcutaneous bDMARDs is either to be administered in weekly dosing, biweekly dosing, or once-monthly dosing. For example, given the half-life of the subcutaneous adalimumab is between 10 and 20 days, the usual frequency for subcutaneous adalimumab administration is once every other week. Therefore, the question regarding the practice of bringing along the medicine while traveling away from home is much more significant and meaningful for drugs with shorter daily dosing intervals as reported and designed in most of the self-reporting questionnaire on assessing medication adherence (Culig and Leppee, 2014; Chung et al., 2015; Wee et al., 2016; Moon et al., 2017). Based on the differences and variation in drug frequencies and intervals among the bDMARDs, item Q9 was appropriate to be included and retained in the SCADS questionnaire. According to the British Society for Rheumatology (BSR), patients should be aware of the need to temporarily discontinue the subcutaneous bDMARDs when having infections (Holroyd et al., 2019). In lieu of this, item Q10 was created to assess on this matter. Identifying IA patients on subcutaneous bDMARDs who deviated biologic treatment injections other than medical instructions given by the prescriber is important to constitute nonadherence within the target population. Compared to other studies on nonadherence among patients on subcutaneous bDMARDs, this item was not assessed within their scope of self-reporting adherence questionnaire (Curkendall et al., 2008; Borah et al., 2009; Li et al., 2010; Bluett et al., 2015; Calip et al., 2017; Mena-Vazquez et al., 2017).

Based on the judgement-quantification of the expert panel on the content validity and the statistical quantification of internal reliability, all the ten items in SCADS appeared to be worthy of being retained for their relevance and importance in measuring subcutaneous bDMARDs adherence among IA patients. In terms of scoring for SCADS, a consensus was achieved among the expert panels. All of them agreed that eight out of ten items were essentials (Q1, Q2, Q4, Q6, Q7, Q8, Q9, and Q10) and should be accounted for the interpretation of adherence. Each correctly answered item contributed one point to the total scores, leading to a possible score range of 0–8. The total score of eight was interpreted as adherence to the subcutaneous bDMARDs prescribed and any score below eight was interpreted as nonadherent. Items Q3 and Q5 were not included in the final score. Both items questioned on patient claiming to inject the bDMARDs according to the prescribed frequency and having their bDMARDs injected on a specific day or date. A patient might falsely claim “yes” for items Q3 and Q5, and hence, scores will only be given if the pharmacist successfully verified the answer in the following items: Q4 and Q6.

Several limitations exist in this study. Since this was a pharmacy-based project, we only included one rheumatologist as the expert panel. The rest of the panels were clinical pharmacists who were involved in rheumatology clinic and academicians involved with clinical pharmacy and rheumatology-related research. Only fifty patients were involved in the initial validation of the tool. This, however, did not affect the reliability result, as our Cronbach’s alpha value was still within a good range despite the small number of patients involved. Due to funding limitation, cross-validation against biologics blood concentration was not conducted in this study. Finally, this tool was only tested and validated in Malay language. Although the English version provided was translated by a bilingual language expert, retesting for reliability prior to its implementation is highly recommended.

Nevertheless, the final version of SCADS offers a systematic score guide to assess bDMARDs adherence among IA patients. This tool should be able to identify the bDMARDs adherence status among IA patients, hence assisting the healthcare professionals in determining whether adherence issues are one of the factors contributing to the failure of bDMARDs therapy. The SCADS tool is also proposed to be used as part of the daily clinical practice during sessions with the R-MTAC pharmacist at the rheumatology clinic. It may serve as a guide to medication counseling and patient education related to bDMARDs therapy to empower bDMARDs adherence among IA patients.

## Conclusion

This study described the development and validation of the subcutaneous biologic disease-modifying antirheumatic drugs adherence score (SCADS) among the IA patients who require the subcutaneous bDMARDs. This final version of the developed questionnaire contains ten items that were comprehensible by the respondent and simple to be answered by using a binary dichotomous response. The instrument was found to be consistent and reliable, with the acceptable Cronbach’s alpha value of 0.707. To our knowledge, this is the first instrument developed to assess adherence among patients currently prescribed with self-injecting subcutaneous bDMARDs.

## Data Availability Statement

The raw data supporting the conclusions of this article will be made available by the authors, without undue reservation.

## Ethics Statement

The studies involving human participants were reviewed and approved by the Research Ethics Committee, Universiti Kebangsaan Malaysia (ETHICS COMMITTEE/IRB REF NO: UKM PPI/111/8/JEP-2017-059). The patients/participants provided their written informed consent to participate in this study.

## Author Contributions

SAR was involved in design and conception of the work, data collection, data analysis, data interpretation, and drafting the article. MMB and MSMS critically revised the article. TCE performed data analysis and interpretation. AMR critically revised the article and approved the final version to be published.

## Conflict of Interest

The authors declare that the research was conducted in the absence of any commercial or financial relationships that could be construed as a potential conflict of interest.

## References

[B1] Abu Samra K. Maghsoudlou A. Roohipoor R. Valdes-Navarro M. Lee S. Foster C. S. (2016). Current treatment modalities of JIA-associated uveitis and its complications: literature review. Ocul. Immunol. Inflamm. 24 (4), 431–439. 10.3109/09273948.2015.1115878 | 26765345

[B2] Aravamudhan N. R. Krishnaveni R. (2015). Establishing and reporting content validity evidence of new training and development capacity building ccale (TDCBS). Management 20 (1), 131–158.

[B3] Bhoi P. Bessette L. Bell M. J. Tkaczyk C. Nantel F. Maslova K. (2017). Adherence and dosing interval of subcutaneous antitumour necrosis factor biologics among patients with inflammatory arthritis: analysis from a Canadian administrative database. BMJ Open 7 (9). e015872 10.1136/bmjopen-2017-015872 | PMC5623530 28928177

[B4] Bluett J. Morgan C. Thurston L. Plant D. Hyrich K. L. Morgan A. W. (2015). Impact of inadequate adherence on response to subcutaneously administered anti-tumour necrosis factor drugs: results from the biologics in rheumatoid arthritis genetics and genomics study syndicate cohort. Rheumatology 54 (3), 494–499. 10.1093/rheumatology/keu358 | 25213131 PMC4334684

[B5] Blum M. A. Koo D. Doshi J. A. (2011). Measurement and rates of persistence with and adherence to biologics for rheumatoid arthritis: a systematic review. Clin. Therapeut. 33 (7), 901–913. 10.1016/j.clinthera.2011.06.001 | 21715007

[B6] Boateng G. O. Neilands T. B. Frongillo E. A. Melgar-Quiñonez H. R. Young S. L. (2018). Best practices for developing and validating scales for health, social, and behavioral research: a primer. Front. Public Health 6, 1–18. 10.3389/fpubh.2018.00149 | 29942800 PMC6004510

[B7] Borah B. J. Huang X. Zarotsky V. Globe D. (2009). Trends in RA patients’ adherence to subcutaneous anti-TNF therapies and costs. Curr. Med. Res. Opin. 25 (6), 1365–1377. 10.1185/03007990902896386 | 19425902

[B8] Calip G. S. Adimadhyam S. Xing S. Rincon J. C. Lee W.-J. Anguiano R. H. (2017). Medication adherence and persistence over time with self-administered TNF-alpha inhibitors among young adult, middle-aged, and older patients with rheumatologic conditions. Semin. Arthritis Rheum. 47 (2), 157–164. 10.1016/j.semarthrit.2017.03.010 | 28410817

[B9] Cattell R. B. (1966). Multivariate behavioral translator disclaimer the scree test for the number of factors. Multivariate Behav. Res. 1 (2), 245–276. 10.1207/s15327906mbr0102 | 26828106

[B10] Cheong A. T. Tong S. F. Sazlina S. G. (2015). Validity and reliability of the Malay version of the Hill-Bone compliance to high blood pressure therapy scale for use in primary healthcare settings in Malaysia: a cross-sectional study. Malays. Fam. Physician 10 (2), 36–44. 27099659 PMC4826579

[B11] Chung W. W. Chua S. S. Mei Lai P. S. Morisky D. E. (2015). The Malaysian Medication Adherence Scale (MALMAS): concurrent validity using a clinical measure among people with type 2 diabetes in Malaysia. PloS One 10 (4), e0124275 10.1371/journal.pone.0124275 | 25909363 PMC4409377

[B12] Culig J. Leppee M. (2014). From Morisky to Hill-Bone; self-reports scales for measuring adherence to medication. Coll. Antropol. 38 (1), 55–62. 24851597

[B13] Curkendall S. Patel V. Gleeson M. Campbell R. S. Zagari M. Dubois R. (2008). Compliance with biologic therapies for rheumatoid arthritis: do patient out-of-pocket payments matter? Arthritis Care Res. 59 (10), 1519–1526. 10.1002/art.24114 | 18821651

[B14] Curtis J. R. Bykerk V. P. Maher A. Schiff M. (2016). Adherence and persistence with methotrexate in rheumatoid arthritis: a systematic review. J. Rheumatol. 43 (11), 1997–2009. 10.3899/jrheum.151212 | 27803341

[B15] Davis L. L. (1992). Instrument review: getting the most from a panel of experts. Appl. Nurs. Res. 5 (4), 194–197. 10.1016/S0897-1897(05)80008-4 |

[B16] Field A. (2013). Discovering Statistics Using IBM SPSS Statistics. London, UK: SAGE Publications.

[B17] Haynes S. N. Richard D. C. S. Kubany E. S. (1995). Content validity in psychological assessment: a functional approach to concepts and methods. Pyschol. Assess. 7 (3), 238–247. 10.1037/1040-3590.7.3.238 |

[B18] Holroyd C. R. Seth R. Bukhari M. Malaviya A. Holmes C. Curtis E. (2019). The British society for rheumatology biologic DMARD safety guidelines in inflammatory arthritis - executive summary. Rheumatology 58 (2), 220–226. 10.1093/rheumatology/key207 | 30137623

[B19] Hughes L. D. Done J. Young A. (2013). A 5 item version of the compliance questionnaire for rheumatology (CQR5) successfully identifies low adherence to DMARDs. BMC Muscoskel. Disord. 14 (1), 1 10.1186/1471-2474-14-286 | PMC3852995 24103582

[B20] Kardas P. Pawel L. Matyjaszczyk M. (2013). Determinants of patient adherence: a review of systematic reviews. Front. Pharmacol. 4, 1–16. 10.3389/fphar.2013.00091 | 23898295 PMC3722478

[B21] Kavanaugh A. Wells A. F. (2014). Benefits and risks of low-dose glucocorticoid treatment in the patient with rheumatoid arthritis. Rheumatology 53 (10), 1742–1751. 10.1093/rheumatology/keu135 | 24729402 PMC4165844

[B22] Klerk E. d. van der Heijde D. Landewé R. van der Tempel H. van der Linden S. (2003). The compliance-questionnaire-rheumatology compared with electronic medication event monitoring: a validation study. J. Rheumatol. 30 (11), 2469–2475. 14677194

[B23] Klerk E. d. Van der Heijde D. Van der Tempel H. Van Der Linden S. (1999). Development of a questionnaire to investigate patient compliance with antirheumatic drug therapy. J. Rheumatol. 26 (12), 2635–2641. 10606375

[B24] Li P. Blum M. A. Von Feldt J. Hennessy S. Doshi J. A. (2010). Adherence, discontinuation, and switching of biologic therapies in medicaid enrollees with rheumatoid arthritis. Value Health 13 (6), 805–812. 10.1111/j.1524-4733.2010.00764.x | 21054657

[B25] Li P. Blum M. A. Von Feldt J. Hennessy S. Doshi J. A. (2010). Adherence, discontinuation, and switching of biologic therapies in medicaid enrollees with rheumatoid Arthritis 13 (6). 10.1111/j.1524-4733.2010.00764.x | 21054657

[B26] Lynn M. R. (1986). Determination and quantification of content validity. Nurs. Res. 35 (6), 382–386. 10.1097/00006199-198611000-00017 | 3640358

[B27] Lyu R. Govoni M. Ding Q. Black C. M. Kachroo S. Fan T. (2016). Treatment persistence among patients with rheumatoid disease (RA, AS, PsA) treated with subcutaneous biologics in Germany. Rheumatol. Int. 36 (1), 143–153. 10.1007/s00296-015-3348-4 | 26314368

[B28] Mahlich J. Sruamsiri R. (2016). Persistence with biologic agents for the treatment of rheumatoid arthritis in Japan. Patient Prefer. Adherence 10, 1509–1519. 10.2147/PPA.S110147 | 27540283 PMC4981174

[B29] Marengo M. F. Suarez-Almazor M. E. (2015). Improving treatment adherence in patients with rheumatoid arthritis: what are the options? Int. J. Clin. Rheumatol. 10 (5), 345–356. 10.2217/ijr.15.39 | PMC4826730 27087857

[B30] Mena-Vazquez N. Manrique-Arija S. Yunquera-Romero L. Ureña-Garnica I. Rojas-Gimenez M. Domic C. (2017). Adherence of rheumatoid arthritis patients to biologic disease-modifying antirheumatic drugs: a cross-sectional study. Rheumatol. Int. 37 (10), 1709–1718. 10.1007/s00296-017-3758-6 | 28631046

[B31] Mikhael E M Hussain S A. Shawky N. Hassali M A. (2019). Validity and reliability of anti-diabetic medication adherence scale among patients with diabetes in baghdad, iraq: a pilot study. BMJ Open Diabetes Res. Care 7 (1), 1–7. 10.1136/bmjdrc-2019-000658 | PMC6626478 31354953

[B32] Moon S. J. Lee W.-Y. Hwang J. S. Hong Y. P. Morisky D. E. (2017). Accuracy of a screening tool for medication adherence: a systematic review and meta-analysis of the morisky medication adherence scale-8. PloS One 12 (11), 1–18. 10.1371/journal.pone.0187139 | PMC5667769 29095870

[B33] Morisky D. E. Ang A. Krousel-Wood M. Ward H. J. (2008). Predictive validity of a medication adherence measure for hypertension cont. J. Clin. Hypertens. 10 (5), 348–354. 10.1111/j.1751-7176.2008.07572.x | PMC2562622 18453793

[B34] Morisky D. E. Green L. W. Levine D. M. (1986). Morisky1986.Pdf. Med Care, 24. 10.1097/00005650-198601000-00007 3945130

[B35] Neubauer S. Cifaldi M. Mittendorf T. Ganguli A. Wolff M. Zeidler J. (2014). Biologic TNF inhibiting agents for treatment of rheumatoid arthritis: persistence and dosing patterns in Germany. Health Econom. Rev. 4 (1), 32 10.1186/s13561-014-0032-4 | PMC4502064 26208932

[B36] Nunnally J. C. Bernstein I. H. (1994). Psychometric Theory. 3rd ed New York: McGraw-Hill.

[B37] Polit D. F. Beck C. T. (2006). The content validity index: are you sure you know what’s being reported? critique and recommendations. Res. Nurs. Health 29 (5), 489–497. 10.1002/nur.20147 | 16977646

[B38] Priest J. Thomas L. Bond S. (1995). Developing and refining a new measurement tool. Nurse Res. 2 (4), 69–81. 10.7748/nr.2.4.69.s8 |

[B39] Rajalingham S. (2018). Novel therapeutic targets in rheumatoid arthritis. Med. Health 13 (1), 12–19. 10.17576/MH.2018.1301.03 |

[B40] Razak S. A. Makmor Bakry M. Redzuan A. M. (2018). Management of rheumatoid arthritis: special consideration for biologic disease-modifying antirheumatic drugs. Asian J. Pharmaceut. Clin. Res. 11 (11), 47 10.22159/ajpcr.2018.v11i11.27953 |

[B41] Salaffi F. Carotti M. Di Carlo M. Farah S. Gutierrez M. (2015). Adherence to anti-tumor necrosis factor therapy administered subcutaneously and associated factors in patients with rheumatoid arthritis. J. Clin. Rheumatol. 21 (8), 419–425. 10.1097/RHU.0000000000000320 | 26587852

[B42] Sandler R. D. Dunkley L. (2018). Osteoarthritis and the inflammatory arthritides. Surgery 36 (1), 21–26. 10.1016/j.mpsur.2017.10.004 |

[B43] Shahrir M. Shahdan M. Mohamed S. Sulaiman W. Mokhtar A. M. Othman M. (2008). Multicentre survey of rheumatoid arthritis patients from Ministry of Health Rheumatology Centers in Malaysia. Int. J. Rheum. Dis. 11 (3), 287–292. 10.1111/j.1756-185X.2008.00379.x |

[B44] Smolen J. S. Breedveld F. C. Burmester G. R. Bykerk V. Dougados M. Emery P. (2016). Treating rheumatoid arthritis to target: 2014 update of the recommendations of an international task force. Ann. Rheum. Dis. 75 (1), 3–15. 10.1136/annrheumdis-2015-207524 | 25969430 PMC4717393

[B45] Smolen J. S. van der Heijde D. Machold K. P. Aletaha D. Landewé R. (2014). Proposal for a new nomenclature of disease-modifying antirheumatic drugs: table 1. Ann. Rheum. Dis. 73 (1), 3–5. 10.1136/annrheumdis-2013-204317 | 24072562

[B46] Smolen J. S. Gladman D. McNeil H. P. Mease P. J. Sieper J. (2019). Predicting adherence to therapy in rheumatoid arthritis, psoriatic arthritis or ankylosing spondylitis: a large cross-sectional study. RMD Open 5 (1), e000585 10.1136/rmdopen-2017-000585 | 30713716 PMC6340591

[B47] Smolen J. S. Landewé R. Breedveld F. C. Buch M. Burmester G. (2014). EULAR recommendations for the management of rheumatoid arthritis with synthetic and biological disease-modifying antirheumatic drugs: 2013 update. Ann. Rheum. Dis. 73 (3), 492–509. 10.1136/annrheumdis-2013-204573 | 24161836 PMC3933074

[B48] Smolen J. S. Landewé R. Bijlsma J. Burmester G. Chatzidionysiou K. (2017). EULAR recommendations for the management of rheumatoid arthritis with synthetic and biological disease-modifying antirheumatic drugs: 2016 update. Ann. Rheum. Dis. 76 (6), 960–977. 10.1136/annrheumdis-2016-210715 | 28264816

[B49] Smolen J. S. Landewé R. B. M. Bijlsma J. W. J. Burmester G. R. Dougados M. Kerschbaumer A. (2020). EULAR recommendations for the management of rheumatoid arthritis with synthetic and biological disease-modifying antirheumatic drugs: 2019 update. Ann. Rheum. Dis. 79 (6), S685–S699. 10.1136/annrheumdis-2019-216655 | 31969328

[B50] Voshaar M. Vriezekolk J. Van Dulmen S. Van Den Bemt B. Van De Laar M. (2016). Barriers and facilitators to disease-modifying antirheumatic drug use in patients with inflammatory rheumatic diseases: a qualitative theory-based study. BMC Muscoskel. Disord. 17 (1), 1–12. 10.1186/s12891-016-1289-z | PMC5075197 27769224

[B51] Waltz C. F. Bausell R. B. (1981). Nursing Research: Design, Statistics, and Computer Analysis. F. A. Davis.

[B52] Waltz C. F. Strickland O. Lenz E. R. (2017). Measurement in Nursing and Health Research: 4th Edn Edited by Joseph Morita and Pamela Lankas. 5th edn. New York: Springer Publishing Company, LLC.

[B53] Wee A. S. Shahrir M. Redzuan A. M. (2016). Medication adherence status among rheumatoid arthritis patients. Int. J. Pharm. Pharmaceut. Sci. 8 (7), 3–7.

[B54] Wynd C. A. Schmidt B. Schaefer M. A. (2003). Two quantitative approaches for estimating content validity. West. J. Nurs. Res. 25 (5), 508–518. 10.1177/0193945903252998 | 12955968

[B55] Zamanzadeh V. Ghahramanian A. Rassouli M. Abbaszadeh A. Alavi-Majd H. Nikanfar A.-R. (2015). Design and implementation content validity study: development of an instrument for measuring patient-centered communication. J. Caring Sci. 4 (2), 165–178. 10.15171/jcs.2015.017 | 26161370 PMC4484991

